# Integrated Transcriptomic and Proteomic Analyses of Antler Growth and Ossification Mechanisms

**DOI:** 10.3390/ijms252313215

**Published:** 2024-12-09

**Authors:** Ruijia Liu, Pan Zhang, Jiade Bai, Zhenyu Zhong, Yunfang Shan, Zhibin Cheng, Qingxun Zhang, Qingyun Guo, Hao Zhang, Bo Zhang

**Affiliations:** 1 State Key Laboratory of Animal Biotech Breeding, Beijing Key Laboratory for Animal Genetic Improvement, College of Animal Science and Technology, China Agricultural University, Beijing 100193, China; s20233040754@cau.edu.cn (R.L.); zhanghao827@163.com (H.Z.); 2 Beijing Milu Ecological Research Center, Beijing Academy of Science and Technology, Beijing 100076, China; baijiade234@aliyun.com (J.B.); zhyzh@milupark.org.cn (Z.Z.); shanyunfang@yeah.net (Y.S.); czb@milupark.org.cn (Z.C.); zhangqingxun1990@126.com (Q.Z.); guoqingyun1987@126.com (Q.G.)

**Keywords:** velvet antler, Chinese milu deer, 4D DIA, antler regeneration, ossification

## Abstract

Antlers are the sole mammalian organs capable of continuous regeneration. This distinctive feature has evolved into various biomedical models. Research on mechanisms of antler growth, development, and ossification provides valuable insights for limb regeneration, cartilage-related diseases, and cancer mechanisms. Here, ribonucleic acid sequencing (RNA-seq) and four-dimensional data-independent acquisition (4D DIA) technologies were employed to examine gene and protein expression differences among four tissue layers of the Chinese milu deer antler: reserve mesenchyme (RM), precartilage (PC), transition zone (TZ), cartilage (CA). Overall, 4611 differentially expressed genes (DEGs) and 2388 differentially expressed proteins (DEPs) were identified in the transcriptome and proteome, respectively. Among the 828 DEGs common to both omics approaches, genes from the collagen, integrin, and solute carrier families, and signaling molecules were emphasized for their roles in the regulation of antler growth, development, and ossification. Bioinformatics analysis revealed that in addition to being regulated by vascular and nerve regeneration pathways, antler growth and development are significantly influenced by numerous cancer-related signaling pathways. This indicates that antler growth mechanisms may be similar to those of cancer cell proliferation and development. This study lays a foundation for future research on the mechanisms underlying the rapid growth and ossification of antlers.

## 1. Introduction

A velvet antler is an organ composed of skin, nerves, blood vessels, cartilage, and other tissues [1]. Owing to its annual regenerative capacity, it serves as a model for regeneration and medical research on cartilage-related diseases [2,3]. In addition, velvet antler grows rapidly, with its maximum growth rate surpassing that of cancer tissue, rendering it an exemplary growth system model for studying cell proliferation and non-carcinogenic differentiation [4,5,6]. Therefore, the growth and development mechanisms of velvet antlers have garnered considerable attention from numerous scholars.

Deer antler growth is primarily regulated by growth centers located at the antler tips [7]. Transcriptomic and proteomic analyses conducted on Gansu red deer (*Cervus elaphus kansuensis*) antlers at different growth stages (30, 60, and 90 days) revealed that genes annotated to the WNT pathway might play pivotal roles in regulating the rapid growth of deer antlers [8]. Proteomic analysis of sika deer antler tips at six different developmental stages indicated that chondrogenesis is a prominent feature during the antler growth phase, whereas the ossification stage is primarily associated with the reconstruction of the bone matrix [9]. By constructing a cellular atlas of sika deer antler regeneration across various stages, the presence of a structure analogous to the blastema-like structure observed in amphibian limb regeneration has been demonstrated during the antler regeneration process. Furthermore, a population of “antler blastema progenitor cells” (ABPCs) capable of self-renewal, osteochondral differentiation, and bone tissue repair has been identified [10]. Although numerous studies have been conducted on the rapid growth of antlers, identifying the key genes that regulate this rapid growth, and ossification remains a crucial aspect of current research on antler growth mechanisms.

According to histological studies, antlers can be divided into four tissue layers, from distal to proximal, which are associated with chondrogenesis: reserve mesenchyme (RM), precartilage (PC), transition zone (TZ), and cartilage area (CA) [11,12,13]. Thus, analyzing gene and protein expression patterns across these multiple tissue layers can offer valuable insights into the potential molecular mechanisms regulating deer antler growth and chondrogenesis.

The Chinese milu deer, also known as Père David’s deer (*Elaphurus davidianus*), is a unique cervid species native to China that was listed as extinct in the wild on the IUCN Red List [14]. However, with the robust protection efforts of the Chinese government, the milu deer population has now surpassed 9000 individuals [15]. The branching pattern of Chinese milu deer antlers is unique, and the natural shedding time of these antlers differs significantly from those of sika deer and red deer [16]. Currently, there have been no reports on transcriptomic or proteomic studies of Chinese milu antler. In this study, we aimed to identify the key genes and pathways that regulate antler growth and chondrogenesis by detecting gene and protein expression levels in the four antler tissue layers (RM, PC, TZ, and CA) of the milu deer.

## 2. Results

### 2.1. Overview of the Transcriptome Sequencing Data

**Figure 1:**
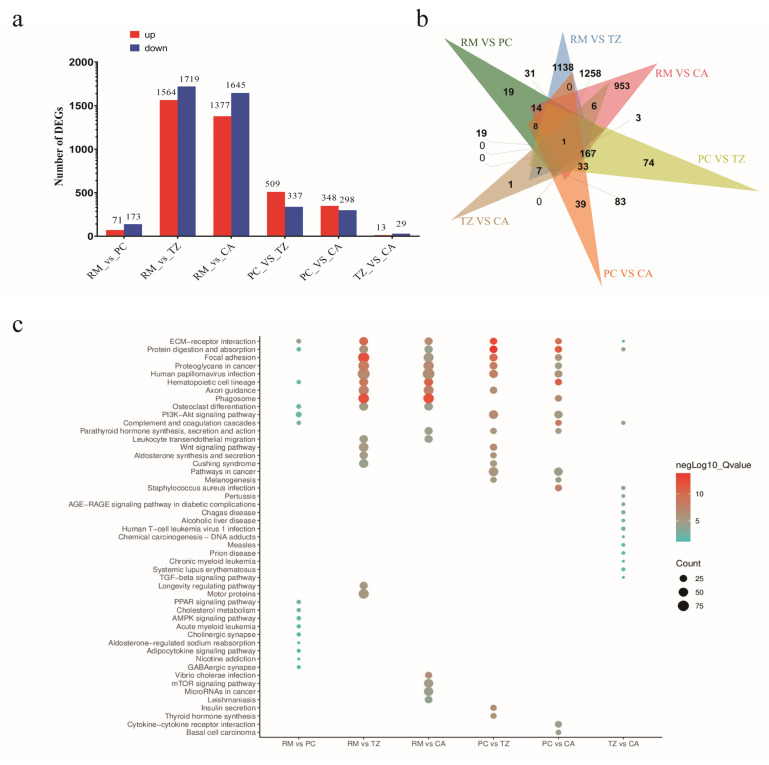
Number of differentially expressed genes (DEGs) and functional enrichment analysis and statistics of DEGs. (**a**) DEGs statistics of 6 groups. (**b**) Overlapping statistics of DEGs obtained by comparing six groups. (**c**) The top 15 most significant pathways enriched in DEGs across the six groups. In all pairwise comparisons, upregulated/downregulated genes refer to those that are upregulated/downregulated in the first group of the comparison. For example, in RM_ vs. _PC, upregulated genes are those that are upregulated in RM and downregulated in PC.

A total of 89.76 Gb of clean transcriptome sequencing data were obtained from 12 samples. The clean data for each sample exceeded 6.07 Gb, with a Q30 base percentage above 92.68% (Table S1). The clean reads of each sample were aligned with the reference genome, resulting in mapping ratios ranging from 82.68% to 86.29% (Table S2). Samples from the four tissue layers (RM, PC, TZ, and CA) were compared pairwise, yielding six groups of differentially expressed genes (DEGs). The numbers of DEGs in each group are shown in Figure 1 The TZ_ vs. _CA group exhibited the lowest number of DEGs (42), whereas the RM_ vs. _TZ and RM_ vs. _CA groups showed the highest counts, with 3283 and 3022 DEGs, respectively (Figure 1a). Overall, only one DEG was common to all six groups, namely, *COL11A1* (Figure 1b). Kyoto Encyclopedia of Genes and Genomics (KEGG) enrichment analysis of DEGs from the six groups revealed enrichment primarily in pathways such as ECM-receptor interaction, PPAR signaling pathway, hematopoietic cell lineage, osteoclast differentiation, protein digestion and absorption, PI3K-Akt signaling pathway, focal adhesion, WNT signaling pathway, and longevity-regulating pathway (Figure 1c).

### 2.2. Short Time-Series Expression Miner (STEM) Analysis of DEGs

**Figure 2:**
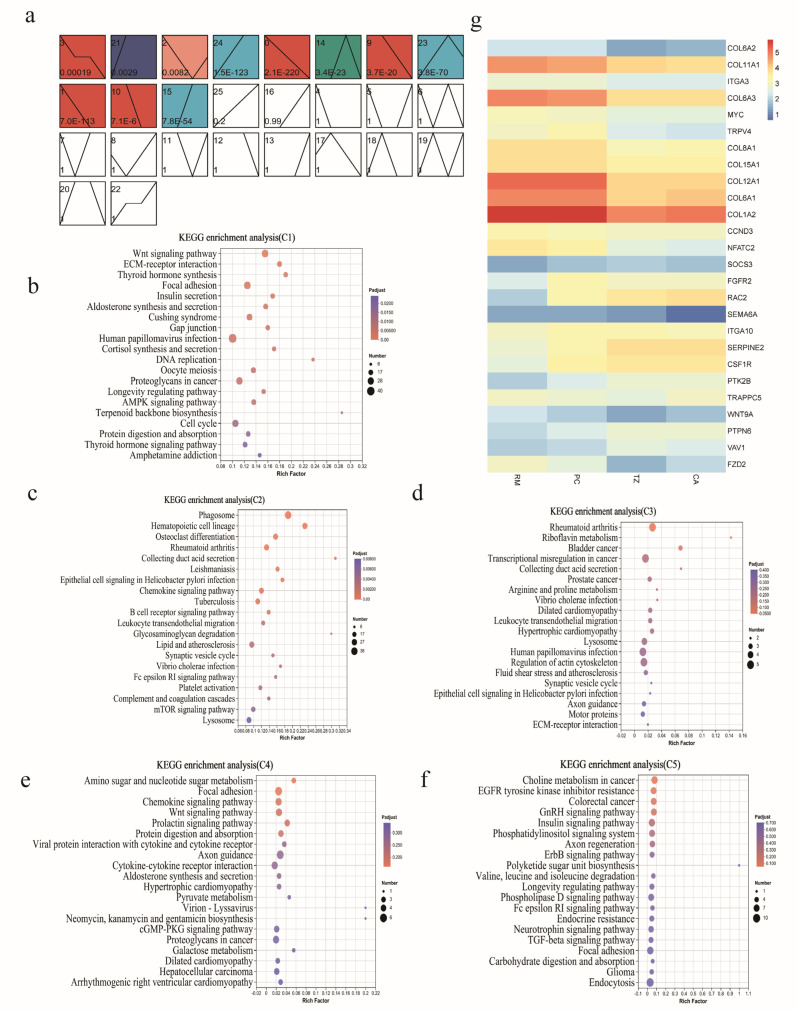
Short Time-series Expression Miner (STEM) analysis was used to explore gene regulatory networks in four tissue layers of velvet. (**a**) The trend analysis of DEGs at different tissue layers. Each profile corresponds to a rectangle, with the number in the top-left corner representing the profile’s ID. The line chart inside shows the trend of expression changes across tissue layers (RM, PC, TZ, CA), and the value in the bottom-left corner is the corresponding significance level (*p*-value). Color-coded trend graphs: These indicate that the time-series pattern of the profile exhibits a significant change trend. Profiles with the same color represent those belonging to the same cluster. Non-color-coded trend graphs: These indicate that the time-series pattern of the profile shows a statistically insignificant change trend. (**b**–**f**) Kyoto Encyclopedia of Genes and Genomics (KEGG) enrichment analysis of DEGs in Clusters 1–5. (**g**) Heatmap of functional DEGs in the 5 clusters. C1: Cluster 1 (Profiles 3, 0, 9, 1, 10); C2: Cluster 2 (Profiles 24, 23, 15); C3: Cluster 3 (Profile 21); C4: Cluster 4 (Profile 2); C5: Cluster 5 (Profile 14).

We performed STEM clustering analysis on all the DEGs, yielding 26 profiles, 11 of which were statistically significant. Based on the gene expression trends across the four tissue layers, 11 significant profiles were categorized into five clusters. Cluster 1 comprised Profiles 3, 0, 9, 1, and 10, while Cluster 2 contained Profiles 24, 23, and 15. Clusters 3, 4, and 5 corresponded to Profiles 21, 2, and 14, respectively (Figure 2a). KEGG enrichment analysis was performed on these five clusters (Figure 2b–f). Given the physiological process of gradual ossification from distal to proximal during antler growth, we focused on pathways associated with the regulation of tissue growth and ossification. In Cluster 1, there was significant enrichment of numerous genes from the collagen family, including *COL6A2*, *COL6A1*, *COL12A1*, *COL15A1*, *COL6A3*, *COL8A1*, and *COL1A2*. Additionally, enrichment was observed in genes related to cell apoptosis, such as *NFATC2* and *MYC*; genes associated with osteoclast formation and proliferation, such as *TRPV4*, *NFATC2*, and the cell cycle protein Ccnd3; and the DEG *ITGA3*, which is involved in regulating the actin cytoskeleton. Cluster 2 enriched pathways include hematopoietic cell lineage, osteoclast differentiation, the mTOR signaling pathway, and complement and coagulation cascades, involving genes such as *VAV1*, *SOCS3*, *CSF1R*, *SERPINE2*, *PTK2B*, and *PTPN6*. Clusters 3, 4, and 5 enriched genes related to the regulation of the actin cytoskeleton: *ITGAD*, *ITGAV*, *ITGAM*, *ITGAL*, *ITGA 2*, *ITGA10*, *FGF1*, *FGFR2*, *Rac2*, *VAV1*, *VAV3*, and *PDGFC*; genes associated with angiogenesis: SEMA6A; osteoclast differentiation-related genes: *ACP5*, *Sema6A*, and *CCL19*; and genes regulating limb development: *WNT9A* and *FZD2* (Figure 2g, Table S3).

### 2.3. Analysis of Protein Expression in Four Tissue Layers

**Figure 3:**
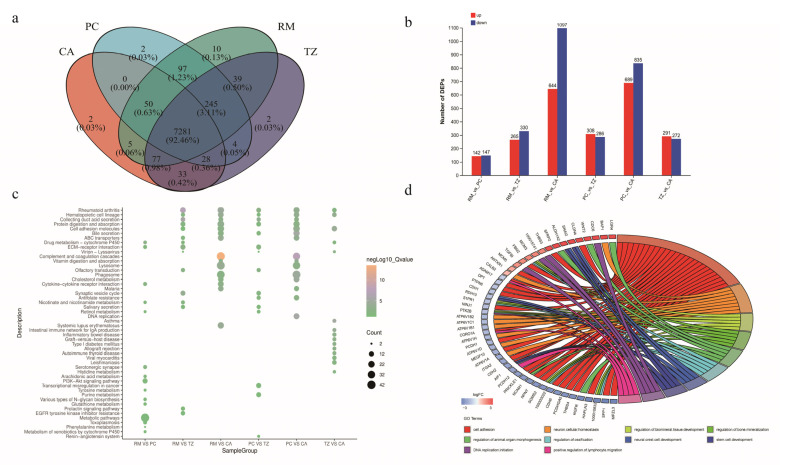
Protein expression characteristics and functional enrichment analysis of differential proteins (DEPs) in the four tissue layers of antlers. (**a**) Venn diagram for protein identification. (**b**) Bar chart of DEPs statistics for the six comparisons. (**c**) KEGG enrichment analysis of DEPs in the six groups. (**d**) Gene ontology (GO) enrichment chord diagram of DEPs in PC vs. CA. In all pairwise comparisons, upregulated/downregulated proteins refer to those that are upregulated/downregulated in the first group of the comparison. For example, in RM_ vs. _PC, upregulated proteins are those that are upregulated in RM and downregulated in PC.

To further identify the genes associated with antler growth and ossification, we used proteomic analysis to investigate protein expression profiles across these four tissue layers. A total of 7916 proteins were identified (Figure 3a), and the statistics for the differentially expressed proteins (DEPs) are shown in Figure 3b. Consistent with the transcriptome data, the PC vs. CA and RM vs. CA groups exhibited the highest number of DEPs. Functional enrichment analysis was performed for these DEPs.

The KEGG pathways enriched by the six groups of DEPs mainly included olfactory transduction, synaptic vesicle cycle, ECM–receptor interaction, protein digestion and absorption, hematopoietic cell lineage, ABC transporters, cell adhesion molecules, and bile secretion. Furthermore, DEPs in the RM vs. PC group showed additional enrichment in numerous metabolic pathways such as glutathione metabolism, nicotinate and nicotinamide metabolism, and several amino acid metabolic pathways. The DEPs in the PC vs. TZ group were enriched in immune-related pathways (Figure 3c).

Gene ontology (GO) enrichment analysis revealed the enrichment of biological processes related to the regulation of animal organ morphogenesis and embryonic limb morphogenesis. The proteins involved were LGR4, CTHRC1, SFRP2, WNT2, SP6, FBN2, ALDH1A2, MEIS2, COL8A1, ID3, ARID5B, RDH10, MEPE, IFRD1, ITGB1BP1, TPPP3, SERPINE2, THBS3, and MTPN. Enrichment was observed for proteins such as SGMS2, PTK2B, and S1PR1, which are involved in the regulation of biological tissue and bone mineralization. Additionally, enrichment was observed for proteins involved in cell differentiation (RAPPC9, SEMA7A, ITGA2, MEGF10, CDK1, and FEM1B) and osteoclast proliferation (NPR3 and TNFSF11). The enrichment of these pathways suggests that the growth and ossification of deer antlers are regulated by multiple pathways (Figure 3d and Figure S1).

### 2.4. Integrated Analysis of Transcriptomics and Proteomics

**Figure 4:**
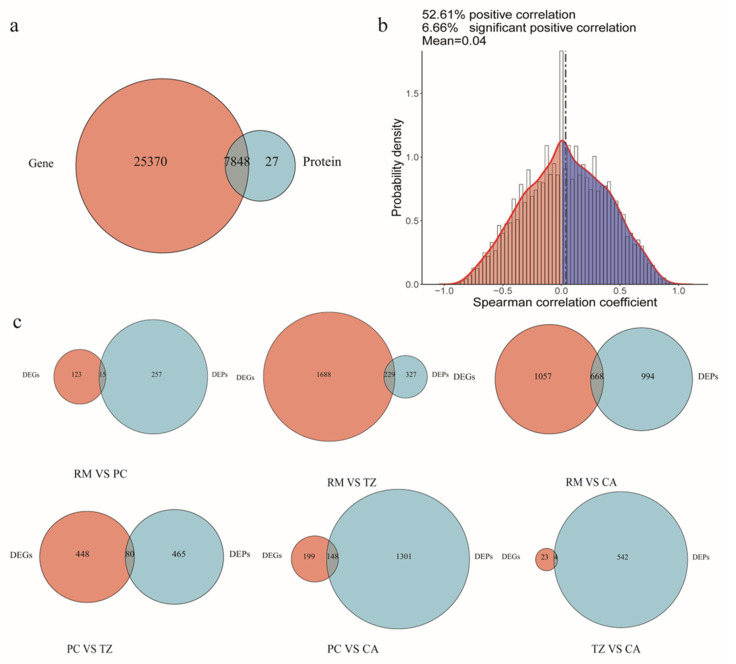
Correlation analysis between transcriptomics and proteomics. (**a**) Overlapping genes between transcriptomics and proteomics. (**b**) Histogram showing gene-wise mRNA–protein Spearman’s correlations. (**c**) Overlapping statistics of DEGs and DEPs.

To further elucidate the genes involved in the regulation of velvet antler growth and ossification, we identified 7848 proteins with the corresponding messenger RNA (mRNA) data (Figure 4a). Spearman’s correlation analysis revealed a moderate correlation between the transcriptome and proteome (rho = 0.442, *p* < 0.01), with 52.61% of the correlations being positive and 6.66% being significantly positive (Figure 4b). The common DEGs identified through the combined transcriptomics and proteomics analysis are shown in Figure 4c.

**Figure 5:**
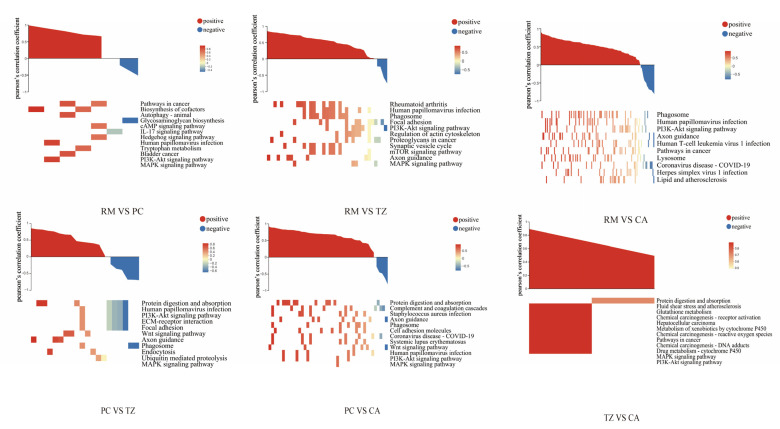
KEGG functional enrichment analysis of overlapping genes between six groups of DEGs and DEPs.

We conducted KEGG enrichment analysis for these genes separately (Figure 5). When comparing RM with PC, we identified key regeneration-related genes *APOD* and *IBSP*, along with several significant pathways: cAMP signaling pathway (*HHIP*), cytokine-cytokine receptor interaction (*IL17D*), pathways in cancer (*HHIP*, *DAPK2*), and biosynthesis of cofactors (*RDH12*, *HAAO*).

In the RM vs. TZ and RM vs. CA comparisons, significant enrichment was observed in pathways such as axon guidance, regulation of the actin cytoskeleton, focal adhesion, the PI3K-Akt signaling pathway, and protein digestion and absorption. Common DEGs between these two groups were *ATP6V1A*, *ATP6V1G1*, *ATP6V1B2*, *ATP6V1C1*, *ATP6V1D*, *SEMA7A*, *PLXNA4*, *SEMA3F*, *THBS3*, *PDGFD*, *ITGA10*, *FGFR1*, *ITGAL*, *TIAM1*, *PDGFD*, *ITGA10*, and *PIK3R1*. Additionally, specific genes, including *NGEF*, *SEMA4D*, *TUBA1A*, *ZYX*, *FLNC*, *SHC3*, *SHC2*, *BCL2L11*, and *Deer_GLEAN_100015035*, were identified in RM vs. TZ. In RM vs. CA, specific genes with differential expression include *ATP1A3*, *NCKAP1L*, *NFATC2*, *ITGA2*, *EPHA8*, *WNT4*, *SEMA6D*, *SCIN*, *SLC7A7*, *SLC7A8*, *PIK3CB*, *SEMA3A*, *COL8A2*, *EFNB1*, *VAV1*, *HSP90AA1*, *KNG1*, *SLC16A10*, *SLC36A2*, *ABLIM3*, *COL12A1*, *COL1A2*, *IGF2*, *ATP1B1*, *COL6A2*, *COL6A1*, *ITGB2*, *NGFR*, *ITGAD*, *SEMA5A*, *RPS6*, *WNT5A*, *COL22A1*, *NTN1*, *SEMA6B*, *SYK*, *SEMA4B*, *BRCA1*, *GNAI1*, *COL27A1*, *EFNA2*, *SPP1*, *CREB3L2*, *RAC2*, *IL2RB*, *SEMA4A*, *ABLIM1*, *PCK2*, and *PKN1*. However, when comparing TZ and CA, only four DEGs were shared between the transcriptome and proteome: *SLC13A5*, *STAB1*, *COL11A1*, and *Deer_GLEAN_100001673*. This suggests the presence of post-transcriptional regulation and that genes without significant differences in expression between the TZ and CA stages may exhibit distinct functionalities. In the combined analysis of PC vs. TZ and PC vs. CA, significantly enriched DEGs were identified in the collagen (*COL8A1*, *COL1A1*, *COL1A2*, *COL9A3*, *COL9A2*, *COL9A1*, *COL12A1*, *COL6A2*, *COL6A1*, *COL11A2*, *COL22A1*, *COL14A1*), integrin (*ITGA11*, *ITGAL*, *ITGA2*, *ITGA10*, *ITGB2*, *ITGAD*), and solute carrier (*SLC7A8*, *SLC16A10*, *SLC36A2*) families. Additionally, we focused on genes involved in the WNT signaling pathway, including *WIF1*, *NFATC2*, *VANGL1*, *WNT4*, *SOST*, *SERPINF1*, *SFRP2*, and *ROR2*.

### 2.5. Verification Transcriptome Data of Quantitative Real-Time Polymerase Chain Reaction (qRT-PCR)

**Figure 6:**
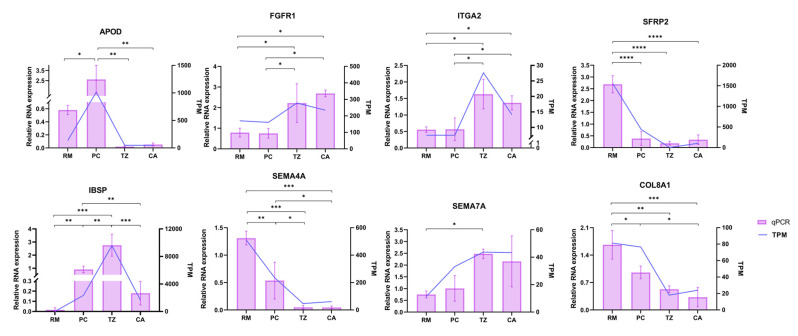
Validation of the DEGs in the four tissue layers of antlers using quantitative real-time polymerase chain reaction (qRT-PCR). * *p* < 0.05, ** *p* < 0.01, *** *p* < 0.001, and **** *p* < 0.0001.

We validated the expression of eight DEGs (*APOD*, *FGFR1*, *ITGA2*, *SFRP2*, *IBSP*, *SEMA4A*, *SEMA7A*, and *COL8A1*) using RT-PCR. By comparing these results with the ribonucleic acid sequencing (RNA-seq) data, we found that the expression trends from qRT-PCR were highly consistent with those from RNA-seq (Figure 6).

## 3. Discussion

In this study, we generated comprehensive transcriptomic and proteomic expression maps for the four tissue layers (RM, PC, TZ, and CA) of the milu deer antler. Data-independent acquisition (DIA) proteomic technology has been extensively employed to identify disease biomarkers because of its extensive protein coverage, high reproducibility, and exceptional accuracy [16,17]. Four-dimensional (4D) proteomics adds a fourth dimension—ion mobility—to traditional proteomics, thereby significantly improving the identification of low-abundance proteins [18,19]. Using the 4D DIA proteomic approach, we identified 7916 proteins in milu deer antlers, a number significantly higher than that identified in the sika deer [20].

GO functional enrichment analysis of DEPs revealed a significant association with biological processes such as organ morphogenesis and embryonic limb morphogenesis. KEGG pathway analysis highlighted enrichment in pathways such as ECM-receptor interaction, protein digestion and absorption, and hematopoietic cell lineage, which were similar to the pathways enriched for DEGs. It is well known that deer antlers undergo seasonal cyclic regeneration, which each year includes the formation of blood vessels, nerves, cartilage, and bone [21,22]. The enriched biological processes and pathways are consistent with the phenotypic characteristics of antler regeneration.

Collagen is the main extracellular matrix (ECM) molecule that supports cell growth and is responsible for organizing and shaping tissues [23]. Various types of collagens are expressed in deer antlers [20], and we identified a large number of differentially expressed collagens in milu deer antlers. Among them, Type I, VI, IX, VIII, XII, XI, XIV, and XXII collagens were differentially expressed in both omics studies. Type I collagen plays a crucial role in regulating the activities of osteoblasts (bone-forming cells), osteoclasts (bone-resorbing cells), and osteocytes (mature bone cells) [24]. It also serves as a guide for the calcification of bone tissue [25]. Type VI collagen inhibits apoptosis and oxidative damage, regulates cell differentiation and autophagy, and promotes tumor growth and progression [26]. Additionally, it is an important bone matrix protein associated with bone remodeling and bone development [27]. As a major component of the pericellular matrix (PCM) in chondrocytes, it can also stimulate chondrocyte proliferation to enhance cartilage tissue generation [28].

In the transcriptome, *COL11A1* was the only DEG common to all six comparisons, and its expression gradually decreased from RM to CA. *COL11A1* is crucial for bone development and collagen fiber assembly [29]. Its upregulation in various cancers suggests that it could serve as a promising biomarker and key player in cancer [30,31]. The growth rate of deer antlers far exceeds that of cancer cells. Current research indicates that gene expression during antler development shows a similar expression profile to that of osteosarcoma, with the developmental mechanisms of antlers adopting molecular strategies used by cancer cells to drive their growth [32,33]. The DEGs we identified were significantly enriched in pathways including “pathways in cancer” and “proteoglycans in cancer,” which supports and corroborates this conclusion.

Integrins are a class of cell adhesion receptors commonly found on the cell surface [34]. Integrins are involved in several critical biological processes, including cell differentiation, adhesion, migration, proliferation, and survival. They also play an indispensable role in tumor angiogenesis [35]. In our combined transcriptomic and proteomic analyses, we identified six integrin family genes (*ITGA11*, *ITGAL*, *ITGA2*, *ITGA10*, *ITGB2*, and *ITGAD*). Integrin α2 (*ITGA2*) is highly expressed in various cancers, and its upregulation promotes tumor proliferation, invasion, migration, and angiogenesis [36]. *ITGA10* is involved in collagen formation through its interaction with the TNC gene and plays a crucial role in cell adhesion, migration, and regulation of inflammatory responses [37,38]. It is also a potential diagnostic and prognostic biomarker for various cancers [39]. *ITGA11*, a receptor for osteonectin, binds to osteonectin to promote WNT pathway activation and osteogenic differentiation of bone marrow stromal cells [40]. *ITGAL* and *ITGB2* are associated with carcinogenesis and immune regulation, and are considered prognostic biomarkers for various cancers [41,42,43]. The extensive identification of genes in the integrin and collagen families highlights the similarities between the growth and development mechanisms of antlers and the molecular mechanisms of cancer cells. Among the DEPs of RM vs. PC, we observed enrichment in pathways related to glutathione metabolism, nicotinate and nicotinamide metabolism, and amino acid metabolism. Active tumor proliferation requires a continuous supply of amino acids for the synthesis of structural and functional proteins, the high material demand, and numerous biochemical reactions in tumor cells [44,45]. The enrichment of these pathways provided new evidence supporting the conclusion that antler growth follows a cancer cell growth and development model [46]. The high expression of these genes in the RM and PC tissue layers further confirms the conclusion that the growth center is located at the tip of the antlers.

The fibroblast growth factor (FGF) family is involved in cartilage development and the regulation of cartilage homeostasis. Studies in mice have shown that *FGF1* knockdown can reduce cartilage damage in osteoarthritis [47]. *FGFR1* is the predominant receptor expressed in human chondrocytes, and it accelerates the degradation of the extracellular matrix in human articular chondrocytes by inducing the expression of transcription factors *RUNX2* and *ELK1* [48]. Studies on *FGFR1* inhibitors have shown that they can effectively protect articular cartilage from the effects of osteoarthritis [49]. In conclusion, this study utilized combined transcriptomic and proteomic analyses to investigate the differences across the four tissue layers of antlers, shedding light on the biological events and gene expression changes involved in antler growth, development, and ossification. These results indicate that the pathways involved in antler growth and development are similar to those involved in cancer cell growth and development. We identified many genes that regulate antler growth, development, and ossification and are associated with pathways such as axon guidance, actin cytoskeleton regulation, protein digestion and absorption, WNT signaling, and cancer-related pathways. Notably, collagen family genes (*COL8A1*, *COL1A1*, *COL1A2*, *COL9A3*, *COL9A2*, *COL9A1*, *COL12A1*, *COL6A2*, *COL6A1*, *COL11A2*, *COL22A1*, *COL14A1*), integrin family genes (*ITGA11*, *ITGAL*, *ITGA2*, *ITGA10*, *ITGB2*, *ITGAD*), signaling molecules (*SEM6D*, *SEMA3A*, *SEMA7A*, *SEMA6B*, *SEMA4A*, *SEMA4B*), and solute carrier family genes (*SLC7A8*, *SLC16A10*, *SLC36A2*) were particularly highlighted for their roles in regulating antler growth and ossification. The combined analysis of transcriptomics and proteomics provides an effective strategy for exploring candidate genes involved in the growth and development of deer antlers and cartilage formation, contributing to research in human regenerative medicine.

## 4. Materials and Methods

### 4.1. Ethics Statement

The animals were reared and handled according to the Guide for the Care and Use of Laboratory Animals in China. All experimental protocols were approved by the Committee on the Ethics of Animal Experiments at the Beijing Milu Ecological Research Center (permit number: 2024-004).

### 4.2. Tissue Collection

In this study, velvet antlers were collected from three free-ranging Chinese milu deer aged 5–6 years from the Beijing Milu Deer Park. Male deer were anesthetized with xylazine hydrochloride (Lu Mian Ning, Beijing, China). After the velvet antlers were sawn off from the antler pedicle, potassium permanganate was immediately applied to stop the bleeding. Subsequently, idazoxan hydrochloride (Lu Xingling, Beijing, China) was injected to revive the Chinese milu deer. Tissue samples were collected from each tissue layer following previously described procedures [31,32]. Three biological replicates were performed for each tissue layer, and a total of 12 samples were collected. The collected samples were frozen in liquid nitrogen and then stored at −80 °C.

### 4.3. RNA Preparation and Sequencing

Total RNA was isolated from velvet antlers using TRIzol reagent (Invitrogen, Carlsbad, CA, USA). The RNA concentration and purity were measured using a NanoDrop 2000 spectrophotometer (Thermo Fisher Scientific, Wilmington, DE, USA). RNA integrity was assessed using an RNA Nano 6000 Assay Kit on an Agilent Bioanalyzer 2100 system (Agilent Technologies, Santa Clara, CA, USA). High-throughput sequencing was performed using the NovaSeq 6000 platform to generate 150 paired-end reads.

### 4.4. Transcriptome Assembly, Annotation, and Differential Expression

After obtaining clean data, we used HISAT2 software (Version 2.1.0) to perform sequence alignment analysis with the Chinese milu deer (Père David’s deer) reference genome (http://animal.omics.pro/code/index.php/Ruminantia/loadByGet?address=ruminantia/Download/GenoDownload.php accessed on 29 March 2024). The read counts were calculated using RSEM (Version 1.3.3), and expression data were normalized to transcripts per million (TPM). Clean data were deposited in the National Center for Biotechnology Information Sequence Read Archive database under accession number PRJNA1149765. The DEGs were identified using DESeq2 algorithm based on the following criteria: |log_2_FC| ≥ 1 and padj < 0.05.

### 4.5. Protein Preparation and DIA Mass Spectrometry Analysis

The 4D-DIA markers were used for quantitative proteomic analysis. Antler samples were ground in liquid nitrogen and lysed with a lysis buffer (8 mol/L urea and 1% SDS). After protein extraction, protein concentration was measured using a BCA Protein Assay Kit (Thermo Fisher Scientific, Wilmington, DE, USA), and protein quality was assessed via SDS-PAGE.

Protein (100 μg) was resuspended in triethylammonium bicarbonate buffer (TEAB) to achieve a final concentration of 100 mM. The mixture was reduced with tris (2-carboxyethyl) phosphine (TCEP) at a final concentration of 10 mM for 60 min at 37 °C and alkylated with iodoacetamide (IAM) at a final concentration of 40 mM for 40 min in the dark at room temperature. After centrifugation at 10,000× *g* at 4 °C for 20 min, the pellet was collected and resuspended in 100 μL of TEAB (100 mM final concentration). Trypsin was added at a 1:50 trypsin-to-protein mass ratio and incubated at 37 °C overnight. Following trypsin digestion, the peptides were dried using a vacuum pump. The dried peptides were resuspended in 0.1% trifluoroacetic acid (TFA), desalted using HLB, and dried again using a vacuum concentrator. Finally, the peptides were quantified using a peptide quantification kit (Thermo Fisher Scientific, Wilmington, DE, USA).

After chromatographic analysis using an EASY-nLC 1200 (Thermo Fisher Scientific, Wilmington, DE, USA), DIA mass spectrometry was performed using a TimsTOF Pro2 mass spectrometer (Bruker Daltonics, Billerica, MA, USA). Tandem mass spectrometry data were processed using the Spectronaut software (Version 14). *p*-values and fold change (FC) for the proteins between the two groups were calculated using the “*t*-test” function in the R package (version 4.3.3). The thresholds of |log_2_FC| ≥ 1.00 and *p*-value < 0.05 were used to identify DEPs. Proteomic data were deposited in the ProteomeXchange Consortium via the iProX Partner Repository with the dataset identifier IPX0009511000.

### 4.6. Functional Annotation of DEGs and DEPs

GO and KEGG enrichment analyses of DEGs and DEPs were performed using the R package clusterProfiler (version 4.14.4). Pathways with a *p*-value < 0.05 were considered significantly enriched.

### 4.7. Protein–mRNA Correlation Analysis

Correlation analyses were conducted between all genes identified via transcriptomics and proteins identified via proteomics, excluding proteins with zero expression in 90% of the samples. The Spearman correlation coefficient (rho) was calculated, and false discovery rate (FDR) values were computed using the Benjamini–Hochberg procedure.

### 4.8. STEM Analysis of the DEGs

Gene expression pattern analysis was performed using STEM software (version 1.3.13). Clustering was based on gene expression correlation coefficients >0.7, with a significance threshold of *p* < 0.05. GO enrichment and KEGG pathway analyses were applied to examine the expression patterns of DEGs in each cluster.

### 4.9. Verification of DEGs

Eight genes (*APOD*, *FGFR1*, *ITGA2*, *SFRP2*, *IBSP*, *SEMA4A*, *SEMA7A*, and *COL8A1*) were selected for qRT-PCR. The primers used for qRT-PCR were designed using Primer Premier software (version 5.0) and are listed in Table S4. The 18S rRNA gene was used as the housekeeping gene. First-strand cDNA was synthesized using a FastKingRT Kit (Tiangen Biotech Co., Ltd., Beijing, China). qRT-PCR was performed using a CFX96 Real-Time System (Bio-Rad, Hercules, CA, USA) with SuperReal PreMix Plus (SYBR Green) (Tiangen Biotech Co., Ltd., Beijing, China). Gene expression levels were calculated using the 2^−ΔΔCt^ method.

### 4.10. Statistical Analysis

Differences in gene expression levels were assessed by one-way analysis of variance (ANOVA) using SPSS software (version 21.0; IBM Corp. Released 2012. IBM SPSS Statistics for Windows, Armonk, NY, USA). Statistical significance was set at *p* < 0.05. Results were presented as mean ± SD.

## Data Availability

The data presented in this study are available in this article and Supplementary Materials.

## Supplementary Materials

The following supporting information can be downloaded at: https://www.mdpi.com/article/10.3390/ijms252313215/s1.

## References

[1] Li C., Suttie J.M. (1994). Light microscopic studies of pedicle and early first antler development in red deer (*Cervus elaphus*). Anat. Rec..

[2] Li C., Zhao H., Liu Z., McMahon C. (2014). Deer antler—A novel model for studying organ regeneration in mammals. Int. J. Biochem. Cell Biol..

[3] Chu W., Hu G., Peng L., Zhang W., Ma Z. (2021). The use of a novel deer antler decellularized cartilage-derived matrix scaffold for repair of osteochondral defects. J. Biol. Eng..

[4] Li X., Liu M., Bai X., Li Y., Zhao Y., Wang S., Wang J. (2019). Molecular cloning, recombinant expression, and purification of osteocalcin in sika deer (*Cervus nippon*) antler. Pol. J. Vet. Sci..

[5] Hu P., Wang Z., Li J., Wang D., Wang Y., Zhao Q., Li C. (2022). IGF1R and LOX modules are related to antler growth rate revealed by integrated analyses of genomics and Transcriptomics. Animals.

[6] Harland W.A. (1971). The problems of atherosclerosis. Practitioner.

[7] Schaefer J.A., Mahoney S.P. (2001). Antlers on female caribou: Biogeography of the bones of contention. Ecology.

[8] Chen Y., Zhang Z., Jin W., Li Z., Bao C., He C., Guo Y., Li C. (2022). Integrative Analyses of Antler Cartilage Transcriptome and Proteome of Gansu Red Deer (*Cervus elaphus kansuensis*) at Different Growth Stages. Animals.

[9] Zhang R., Li Y., Xing X. (2021). Comparative antler proteome of sika deer from different developmental stages. Sci. Rep..

[10] Qin T., Zhang G., Zheng Y., Li S., Yuan Y., Li Q., Hu M., Si H., Wei G., Gao X. (2023). A population of stem cells with strong regenerative potential discovered in deer antlers. Science.

[11] Ba H., Wang D., Yau T.O., Shang Y., Li C. (2019). Transcriptomic analysis of different tissue layers in antler growth Center in Sika Deer (*Cervus nippon*). BMC Genom..

[12] Li C., Suttie J.M. (1998). Electron microscopic studies of antlerogenic cells from five developmental stages during pedicle and early antler formation in red deer (*Cervus elaphus*). Anat. Rec..

[13] Li C., Clark D.E., Lord E.A., Stanton J.A., Suttie J.M. (2002). Sampling technique to discriminate the different tissue layers of growing antler tips for gene discovery. Anat. Rec..

[14] Yang D., Song Y., Ma J., Li P., Zhang H., Price M.R., Li C., Jiang Z. (2016). Stepping-stones and dispersal flow: Establishment of a meta-population of Milu (*Elaphurus davidianus*) through natural re-wilding. Sci. Rep..

[15] Cheng Z.B., Tian X.H., Zhong Z.Y., Li P.F., Sun D.M., Bai J.D., Meng Y.P., Zhang S.M., Zhang Y.Y., Wang L.B. (2021). Reintroduction, distribution, population dynamics and conservation of a species formerly extinct in the wild: A review of thirty-five years of successful Milu reintroduction in China. Glob. Ecol. Conserv..

[16] Heckeberg N.S., Zachos F.E., Kierdorf U. (2023). Antler tine homologies and cervid systematics: A review of past and present controversies with special emphasis on *Elaphurus davidianus*. Anat. Rec..

[17] Li J., Smith L.S., Zhu H.J. (2021). Data-independent acquisition (DIA): An emerging proteomics technology for analysis of drug-metabolizing enzymes and transporters. Drug Discov. Today Technol..

[18] Na L., Xu M., Chen J.L., Chen G.J., Sun J., Zhang Q., Li J.Q., Guo X.L., Zuo Z.F., Liu X.Z. (2023). 4D-DIA quantitative proteomics revealed the core mechanism of diabetic retinopathy after berberine treatment. Eur. J. Pharmacol..

[19] Meier F., Brunner A.D., Frank M., Ha A., Bludau I., Voytik E., Kaspar-Schoenefeld S., Lubeck M., Raether O., Bache N. (2020). diaPASEF: Parallel accumulation-serial fragmentation combined with data-independent acquisition. Nat. Methods.

[20] Chen M., Zhu P., Wan Q., Ruan X., Wu P., Hao Y., Zhang Z., Sun J., Nie W., Chen S. (2023). High-coverage four-dimensional data-independent acquisition proteomics and phosphoproteomics enabled by deep learning-driven multidimensional predictions. Anal. Chem..

[21] Goss R.J. (1995). Future directions in antler research. Anat. Rec..

[22] Gray C., Hukkanen M., Konttinen Y.T., Terenghi G., Arnett T.R., Jones S.J., Burnstock G., Polak J.M. (1992). Rapid neural growth: Calcitonin gene-related peptide and substance P-containing nerves attain exceptional growth rates in regenerating deer antler. Neuroscience.

[23] Ricard-Blum S. (2011). The collagen family. Cold Spring Harb. Perspect. Biol..

[24] Selvaraj V., Sekaran S., Dhanasekaran A., Warrier S. (2024). Type 1 collagen: Synthesis, structure and key functions in bone mineralization. Differentiation.

[25] Shi S.T., Kirk M., Kahn A.J. (1996). The role of type I collagen in the regulation of the osteoblast phenotype. J. Bone Min. Res..

[26] Cescon M., Gattazzo F., Chen P.W., Bonaldo P. (2015). Collagen VI at a glance. J. Cell Sci..

[27] Christensen S.E., Coles J.M., Zelenski N.A., Furman B.D., Leddy H.A., Zauscher S., Bonaldo P., Guilak F. (2012). Altered Trabecular Bone Structure and Delayed Cartilage Degeneration in the Knees of Collagen VI Null Mice. PLoS ONE.

[28] Smeriglio P., Dhulipala L., Lai J.H., Goodman S.B., Dragoo J.L., Smith R.L., Maloney W.J., Yang F., Bhutani N. (2015). Collagen VI enhances cartilage tissue generation by stimulating chondrocyte proliferation. Tissue Eng. Part A.

[29] Kadler K.E., Hill A., Canty-Laird E.G. (2008). Collagen fibrillogenesis: Fibronectin, integrins, and minor collagens as organizers and nucleators. Curr. Opin. Cell Biol..

[30] Bin Lim S., Chua M.L.K., Yeong J.P.S., Tan S.J., Lim W.T., Lim C.T. (2019). Pan-cancer analysis connects tumor matrisome to immune response. NPJ Precis. Oncol..

[31] Vázquez-Villa F., García-Ocaña M., Galván J.A., García-Martínez J., García-Pravia C., Menédez-Rodríguez P., González-del Rey C., Barneo-Serra L., de los Toyos J.R. (2015). COL11A1/(pro)collagen 11A1 expression is a remarkable biomarker of human invasive carcinoma-associated stromal cells and carcinoma progression. Tumor. Biol..

[32] Wang Y., Zhang C., Wang N., Li Z., Heller R., Liu R., Zhao Y., Han J., Pan X., Zheng Z. (2019). Genetic basis of ruminant headgear and rapid antler regeneration. Science.

[33] Jia B.Y., Zhang L.L., Zhang Y.F., Ge C.X., Yang F.H., Du R., Ba H.X. (2021). Integrated analysis of miRNA and mRNA transcriptomic reveals antler growth regulatory network. Mol. Genet. Genom..

[34] Bianconi D., Unseld M., Prager G.W. (2016). Integrins in the spotlight of cancer. Int. J. Mol. Sci..

[35] Ata R., Antonescu C.N. (2017). Integrins and Cell Metabolism: An intimate relationship impacting cancer. Int. J. Mol. Sci..

[36] Huang W., Zhu J., Shi H., Wu Q., Zhang C. (2021). ITGA2 overexpression promotes esophageal squamous cell carcinoma aggression via FAK/AKT signaling pathway. Onco. Targets. Ther..

[37] Afonso J., Coutinho L.L., Tizioto P.C., da Silva Diniz W.J., de Lima A.O., Rocha M.I.P., Buss C.E., Andrade B.G.N., Piaya O., da Silva J.V. (2019). Muscle transcriptome analysis reveals genes and metabolic pathways related to mineral concentration in Bos indicus. Sci. Rep..

[38] Lemma S.A., Kuusisto M., Haapasaari K.M., Sormunen R., Lehtinen T., Klaavuniemi T., Eray M., Jantunen E., Soini Y., Vasala K. (2017). Integrin alpha 10, CD44, PTEN, cadherin-11 and lactoferrin expressions are potential biomarkers for selecting patients in need of central nervous system prophylaxis in diffuse large B-cell lymphoma. Carcinogenesis.

[39] Wang Z.H., Li X.F., Liu S.H., Tang R.M. (2024). ITGA10 can be used as a potential diagnostic and prognostic biomarker of thyroid cancer. Asian J. Surg..

[40] Shen B., Vardy K., Hughes P., Tasdogan A., Zhao Z.Y., Yue R., Crane G.M., Morrison S.J. (2019). Integrin alpha11 is an Osteolectin receptor and is required for the maintenance of adult skeletal bone mass. Elife.

[41] Xu H.S., Zhang A.K., Han X.Y., Li Y.N., Zhang Z.Y., Song L.Y., Wang W., Lou M.Q. (2022). ITGB2 as a prognostic indicator and a predictive marker for immunotherapy in gliomas. Cancer Immunol. Immun..

[42] Li C.Y., Deng T., Cao J.Y., Zhou Y., Luo X.L., Feng Y.L., Huang H., Liu J.H. (2023). Identifying ITGB2 as a potential prognostic biomarker in ovarian cancer. Diagnostics.

[43] Zhang J.C., Wang H., Yuan C., Wu J., Xu J.N., Chen S.Y., Zhang C.H., He Y.L. (2022). ITGAL as a prognostic biomarker correlated with immune infiltrates in gastric cancer. Front. Cell Dev. Biol..

[44] Phan L.M., Yeung S.C., Lee M.H. (2014). Cancer metabolic reprogramming: Importance, main features, and potentials for precise targeted anti-cancer therapies. Cancer Biol. Med..

[45] Wang D., Wan X. (2022). Progress in research on the role of amino acid metabolic reprogramming in tumour therapy: A review. Biomed. Pharmacother..

[46] Li C., Li Y., Wang W., Scimeca M., Melino G., Du R., Shi Y. (2023). Deer antlers: The fastest growing tissue with least cancer occurrence. Cell Death Differ..

[47] Li Y.X., Shu J., Kou N.N., Chen H.B., Guo L.M., Yuan Y., He S.X., Zhao G. (2023). FGF1 reduces cartilage injury in osteoarthritis via regulating AMPK/Nrf2 pathway. J. Mol. Histol..

[48] Xie Y., Zinkle A., Chen L., Mohammadi M. (2020). Fibroblast growth factor signalling in osteoarthritis and cartilage repair. Nat. Rev. Rheumatol..

[49] Xu W., Xie Y., Wang Q., Wang X., Luo F., Zhou S., Wang Z., Huang J., Tan Q., Jin M. (2016). A novel fibroblast growth factor receptor 1 inhibitor protects against cartilage degradation in a murine model of osteoarthritis. Sci. Rep..

