# Supplementary figures and images for: Integrated Transcriptomic and Proteomic Analyses of Antler Growth and Ossification Mechanisms
Source: Int J Mol Sci. 2024 Dec 9;25(23):13215. doi: 10.3390/ijms252313215 (PMC11642661; doi:10.3390/ijms252313215)

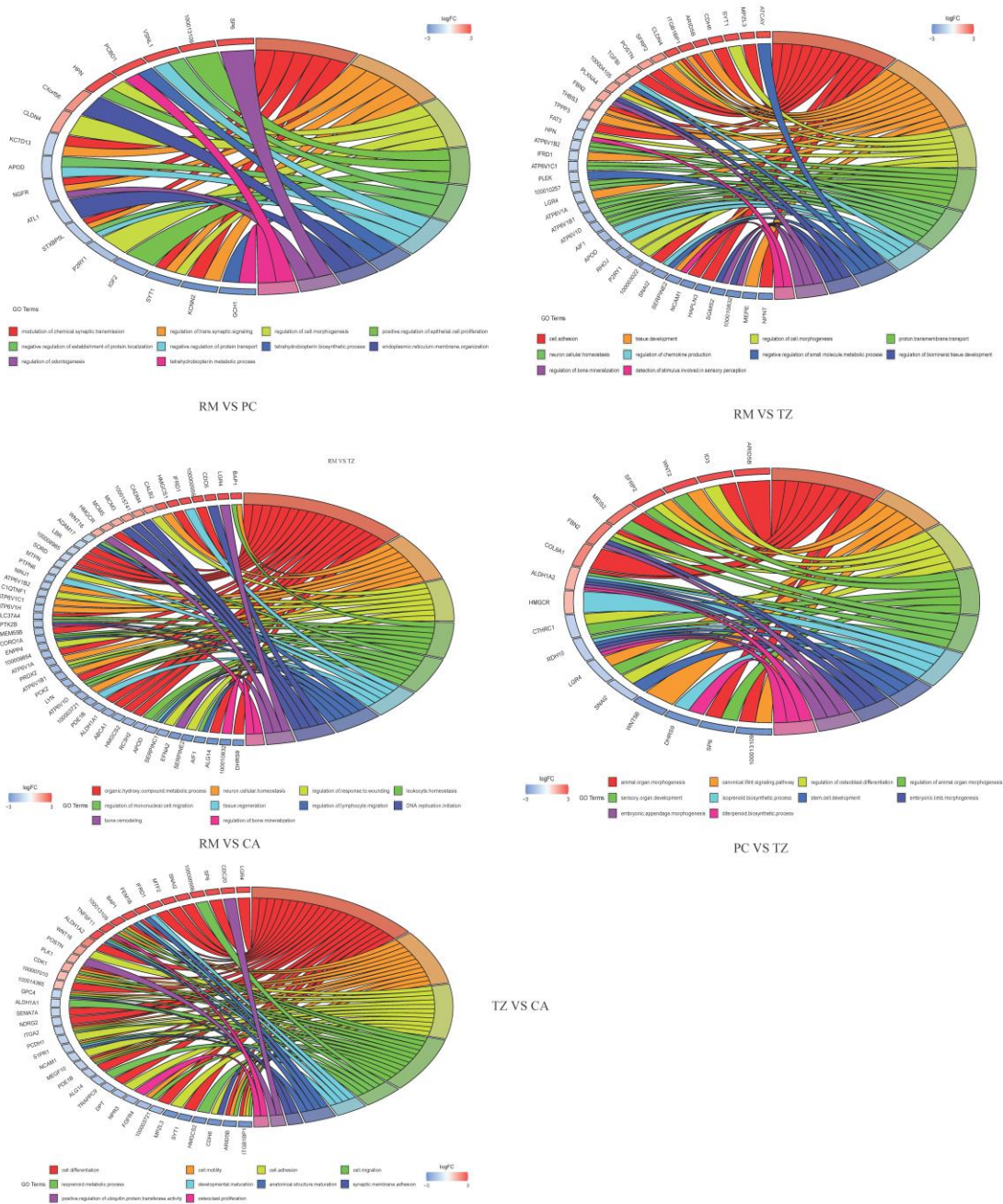

Figure S1: GO enrichment chord diagram of 5 groups of differential expression proteins (DEPs).

Supplement: Supplementary file 1 [file ijms-25-13215-s001.zip › ijms-3302364-supplementary.pdf]
